# Supplementary material for: Glutathione in the nucleus accumbens regulates motivation to exert reward-incentivized effort
Source: eLife. 2022 Nov 8;11:e77791. doi: 10.7554/eLife.77791 (PMC9642999; doi:10.7554/eLife.77791)
Supplement: Transparent reporting form [file elife-77791-transrepform1.docx]

***eLife’s* transparent reporting form**

We encourage authors to provide detailed information *within their submission* to facilitate the interpretation and replication of experiments. Authors can upload supporting documentation to indicate the use of appropriate reporting guidelines for health-related research (see [EQUATOR Network](http://www.equator-network.org/%20)), life science research (see the [BioSharing Information Resource](https://biosharing.org/)), or the [ARRIVE guidelines](http://www.plosbiology.org/article/info:doi/10.1371/journal.pbio.1000412) for reporting work involving animal research. Where applicable, authors should refer to any relevant reporting standards documents in this form.

If you have any questions, please consult our Journal Policies and/or contact us: [editorial@elifesciences.org](mailto:editorial@elifesciences.org).

**Sample-size estimation**

- You should state whether an appropriate sample size was computed when the study was being designed
- You should state the statistical method of sample size computation and any required assumptions
- If no explicit power analysis was used, you should describe how you decided what sample (replicate) size (number) to use

Please outline where this information can be found within the submission (e.g., sections or figure legends), or explain why this information doesn’t apply to your submission:

Sample size was not calculated *a priori* but was based on previous experiments from our lab and similar experiments published in the literature (i.e Bryce and Floresco 2016 Neuropsychopharmacology, Zalachoras et al., 2022 Science Advances). For electrophysiological recordings, sample size was based on previous slice recordings performed in the lab in accumbal medium spiny neurons (MSNs)(Gebara et al., 2021, Biol Psychiatry) and on previous experience with *post hoc* identification of D1- and D2-MSNs through RNAscope analysis. The n numbers are in agreement with the resource equation method to determine the sample size (Charan J, Kantharia ND. 2013 J Pharmacol Pharmacother).

**Replicates**

- You should report how often each experiment was performed (1)
- You should include a definition of biological versus technical replication (2)
- The data obtained should be provided and sufficient information should be provided to indicate the number of independent biological and/or technical replicates (3)
- If you encountered any outliers, you should describe how these were handled (4)
- Criteria for exclusion/inclusion of data should be clearly stated (5)
- High-throughput sequence data should be uploaded before submission, with a private link for reviewers provided (these are available from both GEO and ArrayExpress) (6)

Please outline where this information can be found within the submission (e.g., sections or figure legends), or explain why this information doesn’t apply to your submission:

This information can be found in the main manuscript file in the Statistical analyses section (1). A biological replicate consisted of independent animals (N, for the in vivo part) or individual cells (n, for the electrophysiological recordings) (2). All biological replicate numbers are specified in the figure legends and Source data files (sometimes also in the figures, Figure 6 and Figure 6- figure supplement 6) (3). One outlier was identified with Grubb’s test and removed from the series presented in Figure 6d (4 and 5). No high-throughput sequence data were generated (6).

**Statistical reporting**

- Statistical analysis methods should be described and justified (1)
- Raw data should be presented in figures whenever informative to do so (typically when N per group is less than 10) (2)
- For each experiment, you should identify the statistical tests used, exact values of N, definitions of center, methods of multiple test correction, and dispersion and precision measures (e.g., mean, median, SD, SEM, confidence intervals; and, for the major substantive results, a measure of effect size (e.g., Pearson's r, Cohen's d) (3)
- Report exact p-values wherever possible alongside the summary statistics and 95% confidence intervals. These should be reported for all key questions and not only when the p-value is less than 0.05.

Please outline where this information can be found within the submission (e.g., sections or figure legends), or explain why this information doesn’t apply to your submission:

We provide this information in the main manuscript file in Figure Legend and Statistical analyses section, the Stats table file and the raw data can be found in the Source Data files (1). Raw data is shown for our column plots as individual values (independently of the N) (2). For each experiment we identified the statistical test used, N and other indicated measures as SEM (in Figures, Figure Legend and Stats table) (3) or the exact p-values for all the experiments performed (4).

(For large datasets, or papers with a very large number of statistical tests, you may upload a single table file with tests, Ns, etc., with reference to sections in the manuscript.)

**Group allocation**

- Indicate how samples were allocated into experimental groups (in the case of clinical studies, please specify allocation to treatment method); if randomization was used, please also state if restricted randomization was applied
- Indicate if masking was used during group allocation, data collection and/or data analysis

Please outline where this information can be found within the submission (e.g., sections or figure legends), or explain why this information doesn’t apply to your submission:

Group allocation based on median split is described in the following sections: Results in the subheading section *Individual differences in accumbal GSH levels predict effort-based motivated performance in humans* (page 6), Figure legend 2d and Figure legend 3d.

**Additional data files (“source data”)**

- We encourage you to upload relevant additional data files, such as numerical data that are represented as a graph in a figure, or as a summary table
- Where provided, these should be in the most useful format, and they can be uploaded as “Source data” files linked to a main figure or table
- Include model definition files including the full list of parameters used
- Include code used for data analysis (e.g., R, MatLab)
- Avoid stating that data files are “available upon request”

Please indicate the figures or tables for which source data files have been provided:

Figures with source data files available: Figure 1b-c, Figure1-figure supplement 1d-f, Figure 1d-f, Figure 2d-g, Figure 3d-g, Figure3-figure supplement 3a-k, Figure 4c-k, Figure4-figure supplement 4a-i, Figure 5d-f, Figure 5-figure supplement 5a-j, Figure 6d-e, Figure6-figure supplement6a-d
